# The Connections of Elderly Characters and Taiwanese Society in Edward Yang’s Movie “Yi Yi” (A One and a Two)

**DOI:** 10.1093/geroni/igaa057.335

**Published:** 2020-12-16

**Authors:** Sheng-Chin Hsu

**Affiliations:** Ming Chuan University, Taipei, Taiwan (Republic of China)

## Abstract

As an aging society, Taiwanese is facing the low birthrate and low death rate, and many policies and social systems are facing difficulties. According to the social atmosphere, young and senior groups have many conflicts in between. Finding an alternative approach to reveal the social value of aged people becomes an important mission. The Taiwanese’ movie “Yi-Yi”(A one and a two, 2000) is the final masterpiece of Director Edward Yang, and he won the best director in Festival de Cannes. The story is taken place in a traditional Taiwanese wedding party, and there is a grandma who was invited to this party before her pass out. The grandma did not weak up until the end of this story. The director Yang filmed this sick character in the story and he showed the family members were gathering around their grandma. This study adopts the narrative analysis on elders in “Yi Yi”. There are three findings. First, the elder character is speechless but her sickness drives family members coming home. Second, the long term care is a heavy duty for family, but it reflects the preparations of individual physically and emotionally. Third, the meaning of image of elders is not image itself but family solidarity and social connection. The narrative theory and gerontology build a perspective to understand the social values and narrative functions of elderly people in “Yi-Yi”. It shows the conflict between different age groups and enlarges the spectrum of understanding elders in both Taiwanese’ movie and Taiwanese society.

